# Cost-effective laser metal deposition of 304L stainless steel for repairing and enhancing 316L and mild steel engineering components

**DOI:** 10.1038/s41598-025-19863-1

**Published:** 2025-09-23

**Authors:** Haytham Elgazzar, Hassan Abdel-Sabour, Khalid Abdel-Ghany

**Affiliations:** The Advanced Digital Manufacturing Department, Central Metallurgical Research and Development Institute (CMRDI), Flzzat, Tibbin, 11421 Cairo Egypt

**Keywords:** Additive manufacturing, Laser metal deposition, Stainless steel, Repairing, Energy density, Engineering, Materials science

## Abstract

This study presents a cost-effective additive manufacturing (AM) approach using Laser Metal Deposition (LMD) to enhance the durability and repair of 316L stainless steel and mild steel engineering components. By depositing a protective 304L stainless steel layer, this method extends the components’ life cycle in harsh environments while offering significant cost savings, as 304L powder is less expensive than 316L. The research optimized the LMD process by exploring high scan speeds (up to 8000 mm/min) and powder feed rates (up to 50 g/min) to enhance productivity and ensure an economically viable repair solution. Defect-free layers with strong metallurgical bonding were successfully deposited on both substrates using an optimal energy density of 100–200 J/mm$^2$, an interaction time of 0.5–1.6 seconds, and a powder feed rate of 10–30 g/min. The resulting 304L layers demonstrated enhanced microhardness (around 200 HV) compared to both the 316L and mild steel substrates and corrosion resistance comparable to 316L (and superior to mild steel), with a low corrosion rate of 0.002 mpy in a 3.5% NaCl solution. These results confirm that LMD is a viable and economical solution for repairing and protecting engineering components in various industries such as automotive, pharmaceutical, and marine. The study also highlights the necessary precautions for high-power LMD processes.

## Introduction

Increasing the life cycle and/or repairing the damaged engineering components becomes mandatory for reducing the product’s cost to be more competitive. These components are made from steel alloys such as 316L stainless steel and mild steel due to a good combination of their corrosion and mechanical properties^1,2^. However, these components tend to deteriorate under severe working conditions such as elevated temperatures and corrosive media. Replacing the damaged components is highly expensive, especially for developing countries with low incomes or economic problems. Several solutions have been offered to overcome such problems, through the deposition of a new layer with specific properties for surface protection applications or building up the damaged part from the same manufacturing material. Numerous techniques are currently used such as welding, electroplating, soldering, friction surfacing, laser surface treatment, pulsed laser deposition, cold and thermal spray^3–9^. However, these techniques have various limitations and restrictions, such as less flexibility, high energy consumption, thermal stress, and shape distortion^1,2^. Alternatively, Additive Manufacturing (AM) is a new technology that has rapidly grown in the last decade due to extreme advantages over competitors technologies, such as building complex shapes, flexibility, and material saving^3–7,10,11^. Among various AM processes, the Laser Metal Deposition (LMD) process is a vital process widely used for depositing protective layers against corrosion and repairing engineering components using robotic systems^5–9,12–14^. In this process, a focused laser beam is used to melt the original material in powder on the damaged part, forming a strong metallurgical bond to this part and restoring the original shape. Table 1 shows a comparison of the LMD process with conventional competing processes. As it shown in table 1, the LMD has received significant attention due to its great characteristics, such as material flexibility, where numerous materials and alloys in the form of powders can be used. In addition, high productivity and high precision because of using robots. Moreover, low dilution and distortion of the treated components due to using less energy than other competing processes^15–19^. In addition, table 2 compares the LMD process with Wire Arc Additive Manufacturing (WAAM), both widely used for repair applications^20,21^. The LMD offers key advantages for repairing precision components, such as turbine blades and valve bodies, including lower heat input, reduced distortion, higher precision, and the ability to deposit thin, defect-free 304L stainless steel layers. In contrast, WAAM provides higher deposition rates (up to 10 kg/h) but introduces greater thermal stresses and coarser microstructures, often requiring extensive post-machining. These attributes, combined with the cost-effectiveness of 304L powder (20–30% cheaper than 316L), make LMD a superior choice for applications requiring high-quality surface restoration and minimal thermal impact.

**Table 1:** Comparison of the LMD process with Conventional competing processes.

Characteristic	Laser Metal Deposition (LMD)	Plasma Transferred Arc (PTA)	High Velocity Oxygen Fuel (HVOF)	Ref.
Heat Source	Laser beam	Electric arc	Combustion of oxygen	^22–25^
Temperature	High, localized ($\sim$5,000-10,000$^\circ$C), minimal substrate heating	High ($\sim$10,000-20,000$^\circ$C), moderate substrate heating	Moderate ($\sim$3,000$^\circ$C)	^22,23,25^
Particle Velocity	Low to moderate (deposition via molten pool)	Low to moderate (deposition via molten pool)	High ($\sim$300-800 m/s)	^22,23,25^
Coating Thickness	Thick (0.5-10 mm), suitable for AM and repair	Thick (0.5-5 mm), ideal for surfacing and repair	Thin to moderate (20 $\mu$m-1 mm), ideal for wear-resistant coatings	^22–25^
Porosity	Low(<1%), high-density deposits	Low to moderate (1-5%), depends on parameters	Very low (<1-2%), dense coatings due to high velocity	^22,23,25^
Bond Strength	High, metallurgical bonding due to substrate melting	High,metallurgical bonding due to substrate melting	High,mechanical bonding	^22,23,25^
Material Range	Metals, alloys and composites, precise control for functionally graded materials	Metals, alloys and cermets, suitable for hardfacing	Metals and alloys, limited for refractory materials	^22–25^
Deposition Rate	Moderate(0.1-2 kg/h), slower than PTA	High(1-10 kg/h), efficient for large areas	High (1-5 kg/h), fast for thin coatings	^22,23,25^
Precision	High, excellent for complex geometries and AM	Moderate, good for surfacing but less precise than LMD	Moderate to low, suitable for uniform coatings	^23–25^
Cost	High(expensive equipment, laser systems)	Moderate to high (lower than LMD), higher than HVOF	Moderate(lower equipment and operational costs)	^22,25^
Applications	Aerospace, tool repair, AM, functionally graded coatings	Hardfacing, wear-resistant coatings,turbine blade repair	Wear/corrosion-resistant coatings,turbine components	^22–25^
Environmental Impact	Low(minimal waste, precise material use)	Moderate (some emissions, energy-intensive)	Moderate (fuel combustion, higher energy for gas flow)	^22,23^

**Table 2:** Comparison of the LMD process with Wire Arc AM (WAAM).

Aspect	Laser Metal Deposition (LMD)	Wire Arc Additive Manufacturing (WAAM)	References
Process Description	Laser (fiber/YAG) melts wire/powder, precise layer deposition.	Electric/plasma arc melts wire, rapid layer build.	^20,21,26–28^
Heat Source	Focused laser, small HAZ (<1 mm).	Broad arc, large HAZ (>2 mm).	^20,21,26–29^
Feedstock	wire or powder; wire is cleaner and more cost-effective, powder allows for alloy mixing.	wire which is cost-effective and eliminates powder-related safety concerns.	^21,26–29^
Deposition Rate	1-2 kg/h, ideal for small parts.	5-10 kg/h, suited for large structures.	^21,26,28^
Precision and Resolution	High, smooth finish, complex shapes.	Lower, rough, requires machining.	^26,28^
Material Versatility	Ti, Al, Inconel, 304L,316L powder.	Al, Ti, steel, limited alloys.	^26–29^
Advantages	Precision, low distortion, clean.	High rate, cost-effective, robust.	^21,26–29^
Disadvantages	High cost, slower rate.	Rough finish, high thermal stress.	^26–29^
Applications	Aerospace (blades), medical (implants).	Marine (impellers), construction (bridges).	^21,26,28^
Process Stability	Precise, parameter-sensitive.	Variable, defect risk (porosity).	^26–29^
Environmental Impact	Cleaner with wire feedstock; powder-based LMD poses contamination risks.	Cleaner and safer.	^26–29^

Numerous materials have been used for the LMD process, including 316L and 304L stainless steel, due to their outstanding properties and their wide applications in different industrial sectors^5–7,30^. However, the obtained LMD layers suffer from defects such as pores, residual stresses, and microcracks^1–3,5–7^. These defects originally come from the powder material and/or the improper selection of the process parameters^5,6,9^. In addition, the production rate of the LMD process is relatively low compared with conventional processes, which diminishes the spread of this technology in some industrial sectors^14,31^. Therefore, research on minimizing these defects and increasing the deposition rate is a hot topic. Numerous research efforts have been focused on controlling the microstructure and determining the influence of powder type, shape and process parameters to reduce these defects^5–7,17,18^. Sun et al.^14^, repaired 316L stainless steel using two different commercial types of 316L powders. The investigation results indicated that the powders whose chemical content is close to that of the substrate tend to enhance the properties of the repaired parts. Jeong et al.^32^, studied the effect of substrate properties, mainly yield strength and grain size, on the residual stress of the 316L stainless steel layers deposited on heat-treated and non-treated 316L stainless steel substrates. The results showed the substrate grain size and yield strength reduce the residual stress of the deposited layers. Bernauer et al.^33^, investigated the influence of thermal process conditions and the building height on the thermal history, microstructure, and mechanical properties of 316L stainless steel layers deposited on pre-heated and non-heated 316L stainless steel substrates using wire-fed 308L stainless steel LMD process. The results showed variation in the layer properties according to substrate conditions, which could be used to control layer properties better to apply to specific applications. Errico et al.^34^, deposited. 316L stainless steel layers using the LMD process on the 316L stainless steel components made by selective laser melting to improve the surface properties of these components. Errico showed that the deposited 316L stainless steel layers have better surface roughness. In addition, the microhardness of the treated components improved due to grain refinement that resulted from the LMD process. Callanan et al.^35^, used an electron beam process for repairing 308L stainless steel. The study focused on investigating the response of the repaired parts to high strain-rate dynamic testing. The results showed that the overall dynamic response of the repaired parts and base materials is equivalent. Post-processing such as heat treatment is considered one of the strategies that successfully reduces the defects in the deposited layers. Oh et al.^36^, used in-situ substrate heating and in-situ post-heating to minimize the interfacial cracks that occur on the layer interface. Liu et al.^37^. reported that using the LMD process followed by the hot deformation process for repairing 316L stainless steel significantly enhanced the microstructure and mechanical properties of the repaired parts. Kim et al.^38^, showed that the post-processing using conventional machining and induction-assisted machining enhanced the surface roughness, microstructure, and microhardness of the produced 304L layers by the LMD process. Several strategies have been developed to increase the production rate and enhance the process stability. Gao et al.^39^. developed a new analytical method to simulate the gas-powder flow for a four-port nozzle to obtain uniform and stable powder flows at a high deposition rate. Numerical modeling has been developed for the LMD process to control the process parameters and, therefore, enhance the quality of the product. Zhan et al.^40^. developed a new model to predict the thermal behavior in the molten pool during the LMD repairing of 316L stainless steel. The results showed significant agreement between the developed model and the real part. Peng et al.^41^, developed a molecular dynamics simulation model to investigate the microstructure evolution mechanism of 316L stainless steel during the solidification process of the LMD process. The developed model showed that the LMD process has an evident effect on repairing 316L stainless steel components. The investigation showed the mechanical properties of the repaired components were significantly enhanced due to grain refinement through the solidification process. The above mentioned studies focused on repairing the 316L stainless steel components using 316L stainless steel powder to ensure that the treated components have the same properties, and by controlling the process parameters, common defects such as porosity could be eliminated. In addition, a high deposition rate required a sophisticated design of the deposition head and specific powder properties with good control of processing parameters. Controlling the process parameters might be economical, as it does not require expensive tools. Hence, repairing the damaged 316L stainless steel parts using low-cost material with proper control of the process parameters is becoming mandatory to minimize the overall cost of the repairing process. However, limited efforts have been made to use low-cost materials and /or a high deposition rate to reduce the time and cost of the LMD process. The deposition of low-cost 304L stainless steel protective layers is highly effective in terms of time, energy, and money compared to the cost of replacing the damaged parts. 304L stainless steel is a low-cost material due to the absence of Mo in its composition, has exceptional corrosion resistance with significant mechanical properties close to 316L stainless steel, and is widely used for various industries due to these vital characteristics, making it an ideal material for this study. Deposition of 304L stainless steel layers using the LMD process has been of interest due to its excellent corrosion resistance and appropriate mechanical properties. Melia, et al.^5^, reported that the deposition of 304L stainless steel using the LMD process greatly enhanced the corrosion resistance compared to the wrought 304L stainless steel plate by minimizing the defects such as porosities and using a high solidification rate or reducing micro-segregation at retained $\delta$/$\Upsilon$ interfaces. Wang et al.^6^, investigated the influence of the heat input on the mechanical properties of AISI 304L stainless steel made by LMD process. The results showed the lower heat input yield a finer microstructure, which enhanced the yield and tensile strengths compared with specimens made with the higher heat input. Ning et al.^19^, repaired the 304 stainless steel using intensive high-strength martensitic stainless steel powder by the LMD process. The results indicate that proper control of process parameters such as laser power, scan speed, and powder feed rate are important for obtaining defect-free layers. Table 3 presents a summary of the improved properties of AISI 304L and 316L stainless steel fabricated by the LMD process, as reported in the literature. Although these studies reported the optimum process parameters and their influence on improving corrosion resistance and mechanical properties, limited efforts have been given to the influence of interaction time and minor process parameters.

**Table 3:** A summary of the improved properties of AISI 304L and 316L stainless steel fabricated by the LMD process.

Ref.	Process Parameters	Target Properties	Substrate Material	Deposited Material
^5^	Laser power, scan speed, powder feed rate	Corrosion resistance improved	304L	304L
^6^	Laser power, scan speed, powder feed rate	Yield/tensile strength enhanced	304L	304L
^7^	Laser power, scan speed	Hardness, tensile properties improved	304L	304L
^12^	Laser power, scan speed, powder composition	Microstructure, mechanical properties	316L	316L
^17^	Laser power, scan speed, powder feed rate	Defect-free layers, strength improved	304	Martensitic
^21^	Laser power, thermal conditions	Hardness, tensile properties varied	316L	316L (wire-fed)

While previous studies primarily focused on repaired 316L stainless steel using 316L stainless steel powder at low deposition rates, this study introduces a cost-effective approach by depositing 304L stainless steel layers at high deposition rates and scan speeds, reducing material and operational costs while improving productivity for repair applications. The relations between laser power, speed, interaction time, and powder feed rate were extensively investigated. It is believed that the results of this study will offer an economical solution for protecting expensive engineering components with wide applicability in developed countries.

## Experimental details

### Material preparation

**Fig. 1:**
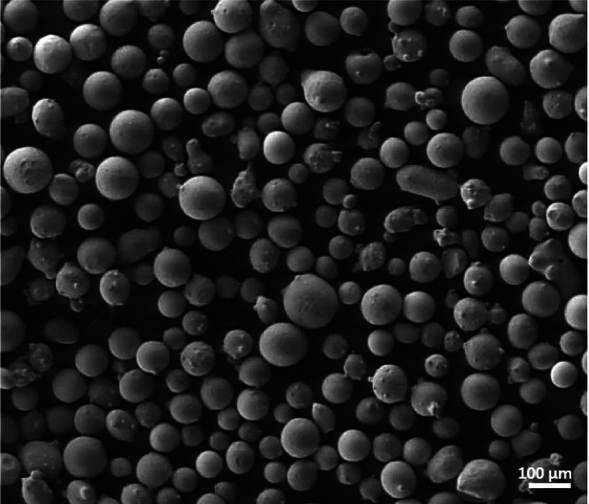
The powder shape of stainless steel 304L.

A commercial 304L stainless steel alloy with average particle sizes between 45 to 120 $\mu$ was used as the deposited powder material. This powder material is less expensive compared to 316L stainless steel and offers comparable properties to 316L stainless steel powder, which makes it an ideal solution for repairing 316L stainless steel components. The powder material can be stuck in the hopper or deposition head depending on the powder grain’s shape, size, and moisture. Generally, gas-atomized powder with a spherical shape is recommended for deposition. An SEM image of the 304L powder is shown in Fig.1, illustrating its predominantly spherical morphology resulting from gas atomization. This spherical shape enhances powder flowability and ensures consistent deposition during the LMD process, contributing to defect-free layers and reliable metallurgical bonding. However, the supplied powder material may contain an irregular powder shape and lower or over grain size powder than the recommended size, leading to powder stuck during deposition. In addition, moisture in the powder accumulates the powder and may lead to being stuck in the hopper even with the continuous stirrer. Therefore, powder screening analysis and heating of the powder for several hours must be implemented before the deposition process. In this regard, the powder material is screened to avoid unwanted sizes and heated at 90 $^o$C for four hours in the oven.

A stainless steel 316L substrates and mild steel were used as the target material. The surfaces of mild steel specimens were ground with emery paper to remove the oxide scale and rinsed with acetone before starting the LMD process. The chemical composition of 304L deposited materials, 316L stainless steel and mild steel substrates are shown in Table 4.

**Table 4:** The chemical composition % of deposited materials and substrates.

Material /Composition	C	Si	Cr	Ni	Mn	P	O	N	F**e**
304L Powder	0.02	0.9	18.4	11.2	1.8	0.035	<0.01	0.08	Bal.
316L Substrate	0.02	0.57	16.2	10.7	1.7	0.040	<0.01	0.09	Bal.
Mild Steel Substrate	0.11	0.16	0.03	0.01	1.29	0.012	0.02	0.01	Bal.

### The Laser Metal Deposition (LMD) process

**Fig. 2:**
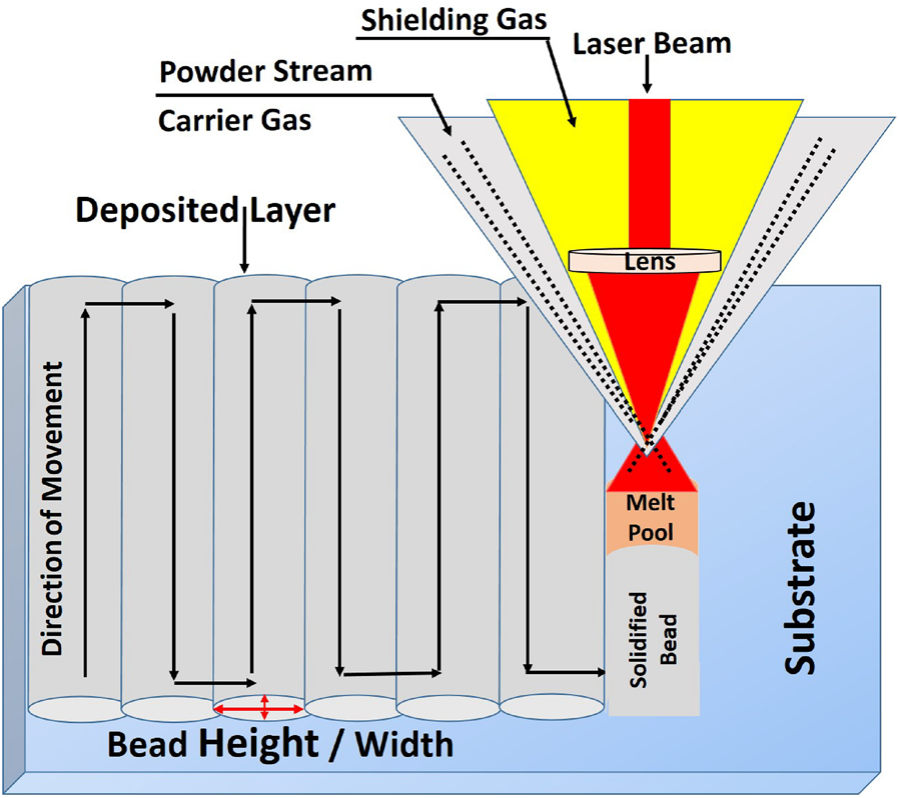
Illustration of the LMD process.

**Fig. 3:**
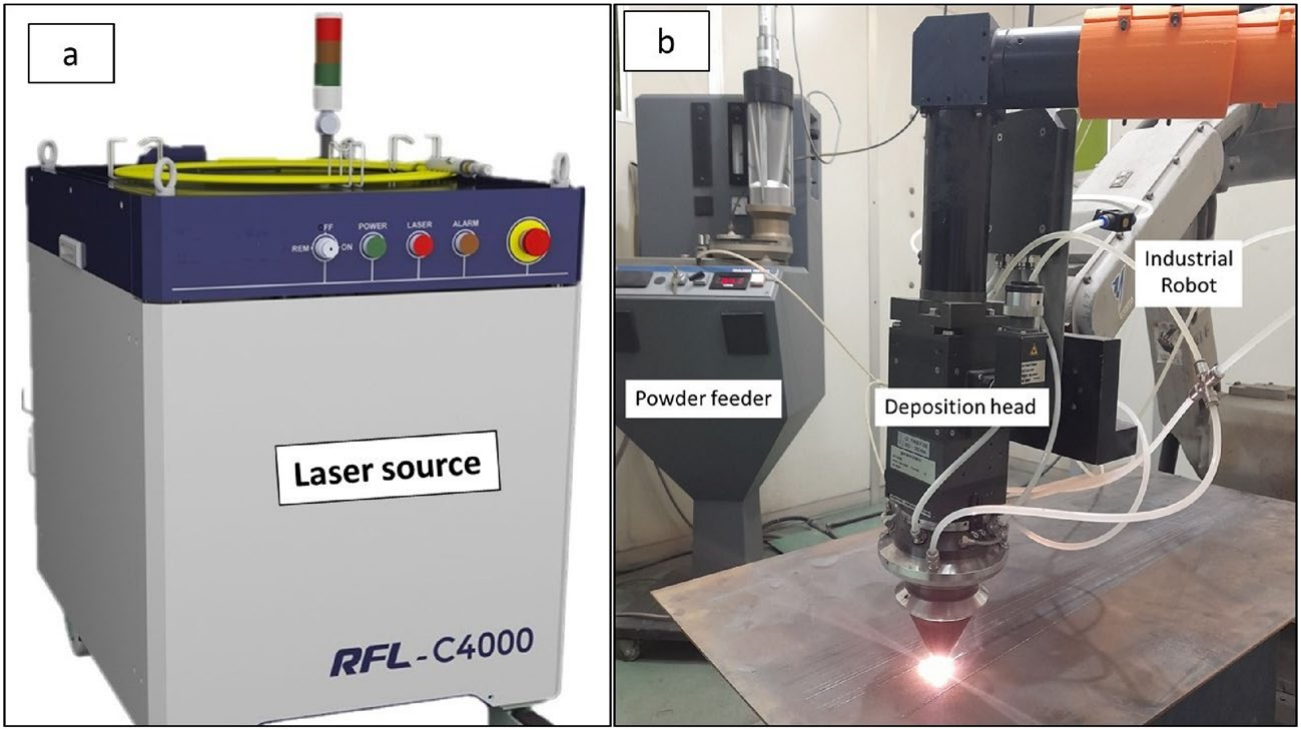
The LMD experimental setup, (**a**) Fiber laser source, (**b**) Industrial robot with powder feeder and deposition head.

The LMD process is illustrated in Fig.2 and the experimental setup applied is shown in Fig. 3. The process was carried out using the 3KW continuous wave fiber laser ($\lambda$=1070 nm, Raycus Co., China) equipped with a coaxial deposition head (YC-50M, Precitec Co.,) and powder feeder (Twin 10C, Sultzer-Metco Co.,). The powder feeder carries the powders to the deposition head through the nitrogen gas. A six-axes robot (Motoman, Yaskawa Co.,) is used for the deposition process.

**Fig. 4:**
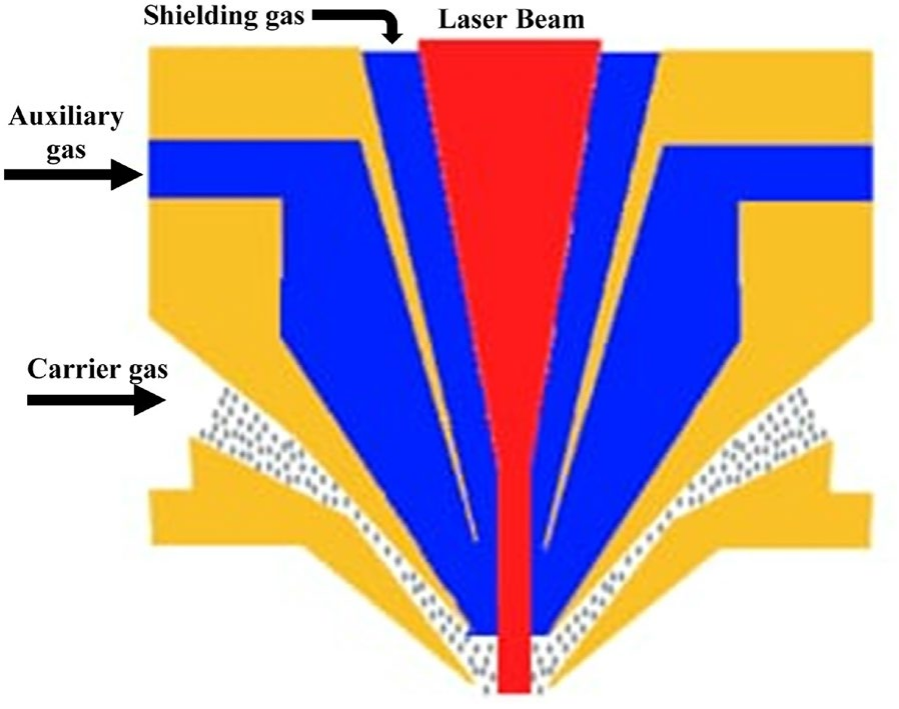
Illustration of the coaxial deposition head and the LMD process gases.

The laser power, spot size and scan speed of the laser beam, and powder feed rate were varied to investigate their influence on the layer dimensions, microstructure, porosity, and crack susceptibility. The gases used for the LMD process are classified into shielding gas, auxiliary gas, and powder carrier gas and are illustrated in Fig. 4.

Generally, shielding gases such as nitrogen and argon are used for cooling, protecting the lens during the process from spatters and protecting the melt pool from oxidation by forming a shielding curtain.

Nitrogen is the gas most commonly employed due to its low chemical activity and cheapness compared with argon. Usually, the auxiliary gas and carrier gas are the same as the shielding gas. Different parameters, such as the auxiliary, carrier, shielding gases flow rate, standoff distance, and rotating speed of the powder feeder must be optimized before processing. The auxiliary gas is required to maintain the velocity of the powder particle at the nozzle exit and to be equal to the carrier flow to guarantee the stability of the LMD process^42^. While shielding gas is essential to protect the melt pool from oxidation. The standoff distance is the distance between the deposition head nozzle exit and the substrate surface where the powder stream shape is constant at this distance.

Nitrogen gas was used as the powder carrier and shielding gas to protect the deposition process against oxidation, the gas flow rates for all experiments were 5 l/min. The rotating speed of the disk-type powder feeding system is responsible for determining the quantity of powder materials delivered to the deposition head and subsequently molten pool. The rotating speed can be varied from 5 to 90 RPM, which changes the powder feed rate from 2 to 50 g/min. A series of experiments described below were made to optimize these parameters, as it is vital to guarantee the results of the LMD process. The LMD process is complex because it depends on various parameters such as laser power, interaction time between laser beam and powder materials, scanning speed, and powder feed rate. Thus, it is essential to compromise all these parameters to get defect-free layers with solid metallurgical bonding between the deposited layers and the substrates. The experiments were designed to combine all the LMD parameters and corresponding features, such as bead height, width..etc. The laser power, scan speed, and powder feed rate and interaction time significantly affect layer characteristics and properties among various processing parameters. The processing parameters can be expressed by laser energy density [E] which is the applied energy per unit area, and can be calculated by the eq.1^13^:

1 $$\begin{aligned} E = (P / d * v)\quad [J/mm^2] \end{aligned}$$

Where P is the laser power [watt], d is the beam diameter [mm], and v is the scanning speed [mm.s-1]. While the interaction time (T) was calculated by the eq.2^13^:

2 $$\begin{aligned} T= d / v\quad [sec] \end{aligned}$$

Accordingly, variable processing parameters were changed to obtain a flexible processing window that may varied according to the desired applications such as crack repairing or deposition of protective coatings. Table 5 shows the variable processing parameters used to achieve the maximum productivity of the LMD system and the surveillance of the LMD process stability.

**Table 5:** The main LMD processing parameters.

Laser Power	Scan Speed	Spot Size	Powder Feed Rate	Energy Density	Interaction Time	Gas flow rate
[W]	[mm/min]	[mm]	[g/min]	[J/mm$^2$]	[sec]	[l/min]
1000 to 3000	100 to 8000	4	10 to 50	50 to 300	0.2 to 2.4	5

### Quality evaluation

All specimens were extensively investigated and evaluated using visual inspection and various characterization techniques with respect to layer shape, dimensions (width and height), and microstructures. To ensure reproducibility and consistency of results across the wide parameter window explored in this study, each unique combination of process parameters (laser power, scan speed, and powder feed rate, as outlined in Table 5) was tested a minimum of three times. For each replicate, single beads were deposited on 316L stainless steel or mild steel substrates, and their dimensions (width and height) were measured using a Vernier caliper micrometer. Only parameter sets producing consistent ”good” beads (continuous and defect-free) across all trials were selected for further analysis. The deposited beads were inspected visually and rated good or bad according to their shape and dimensions. The visual inspection is vital in this study as it eliminates the bad specimens and saves the time needed to investigate other successful specimens. The morphology and microstructures were investigated using optical and scanning electron microscopes. For the microstructure observation, all specimens were cut transversally, grounded, polished in diamond paste, and etched with reagent 40 ml $\hbox {H}_2$O +40 ml HNO3+ 20 ml HF. The phases present in the layers were determined by X-ray diffraction (XRD) using CuK$\alpha$ radiation. Corrosion measurements of the optimum specimens were measured using (IM6, Zahner, Germany). Corrosion experiments were performed at room temperature in aqueous 3.5 wt.% NaCl solution using a three-electrode electrochemical cell. The specimen was the working electrode, a Pt wire served as a counter electrode, and a standard calomel electrode (SCE) was used as a reference electrode. The exposed area of the sample was 0.3 cm$^2$. The examined specimens were first immersed in the test solution for 15 min to stabilize the open circuit potential (OCP). Subsequently, an anodic potentiodynamic polarization was performed starting from the cathodic zone, 0.3 V below the OCP, and increasing into the anodic zone at 1mV/s scan rate.

## Results and discussion

### The powder material stream optimization

**Fig. 5:**
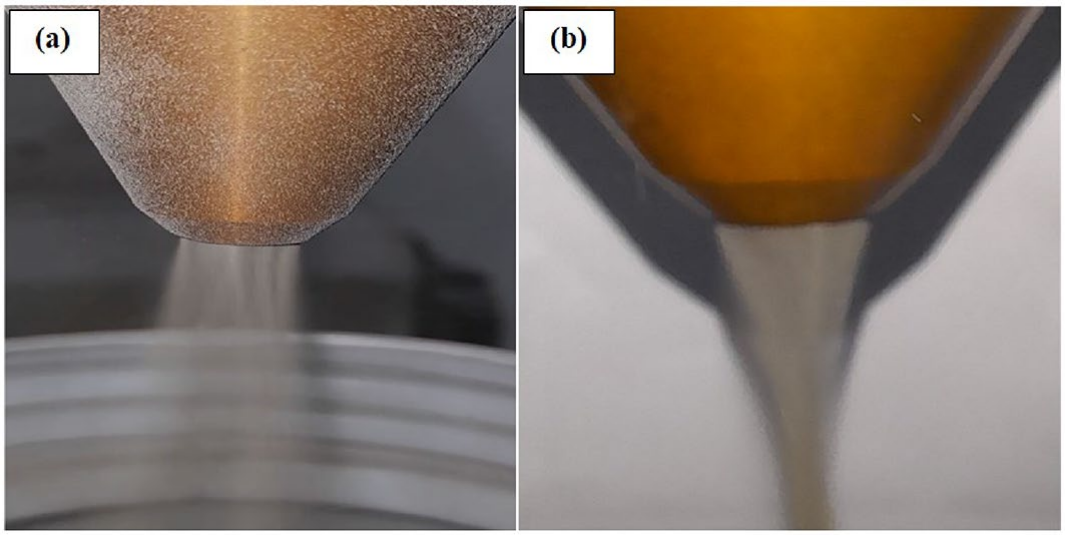
The powder stream shape (**a**) before adjustment and (**b**) after adjustment.

The efficiency of the LMD depends on powder stream control, where a well-concentrated powder in the stream is needed to obtain the desired quality and avoid waste of powders^43^. With a high powder feed rate, the powder stream loses its concentration rapidly compared with a low powder feed rate, which influences the deposited layer dimensions^44^. On the other hand, adjusting the gas flow setting influences the powder concentration in the stream in the processing area to increase the efficiency of the LMD process. Hence, adjusting the powder stream was done by optimizing the gas flow setting. In the LMD process, a coaxial nozzle is composed of three gas channels. The first gas is a carrier gas that holds the powder particles inside the nozzle until they get to the molten pool. The second is shielding gas that protects the molten pool from oxidation, and the third is auxiliary gas used to shape the powder stream. The trajectory and concentration of the powder in the stream were adjusted by a combination of control of the three gases to obtain a constant stream. The shielding gas pressure was incrementally adjusted up to 1.5 bar to evaluate its influence on the powder stream while maintaining the carrier gas flow at a constant rate of 5 L/min. Fig.5., shows the powder stream’s behavior before and after optimizing the process gases, with the powder feed rate fixed at 10 g/min. It found that the shielding gas plays a key role in focusing the powder within a confined area. However, when the shielding gas pressure reached or exceeded 1 bar, it caused the powder particles to disperse away from the laser spot. Consequently, a shielding gas pressure of 0.5 bar was selected, as it consistently confined the powder to a narrow area, facilitating effective melting of most powder particles during the deposition process.

### Controlling Bead width

**Fig. 6:**
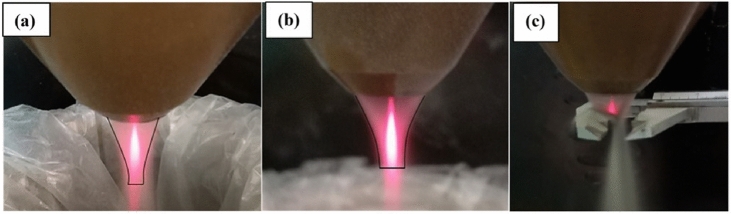
(**a**) Auxiliary gas off (**b**) Auxiliary gas on (**c**) Measuring the powder spot size.

Usually, the protective layers consist of several single beads that overlap each other. Therefore, it is important to focus on optimizing the single bead shape and dimensions.The focal point of the powder stream is established at a distance of 12 mm from the deposition head’s tip, according to the specifications of the head. At this focal point, the powder spot’s width was noted to expand from 3.4 mm to 4.65 mm, as determined through measurements with a Vernier caliper micrometer, as shown in Fig.6(a-c) with the activation of the auxiliary gas function. This adjustment enables the creation of either wide or narrow beads tailored to specific application needs. A wider bead is preferable for applications such as corrosion protection or crack repair, as it minimizes both the time and cost associated with the LMD process. Conversely, beads with a narrower width and greater thickness are more suitable for reconstructing damaged components, such as gear teeth or 3D printing applications.

**Fig. 7:**
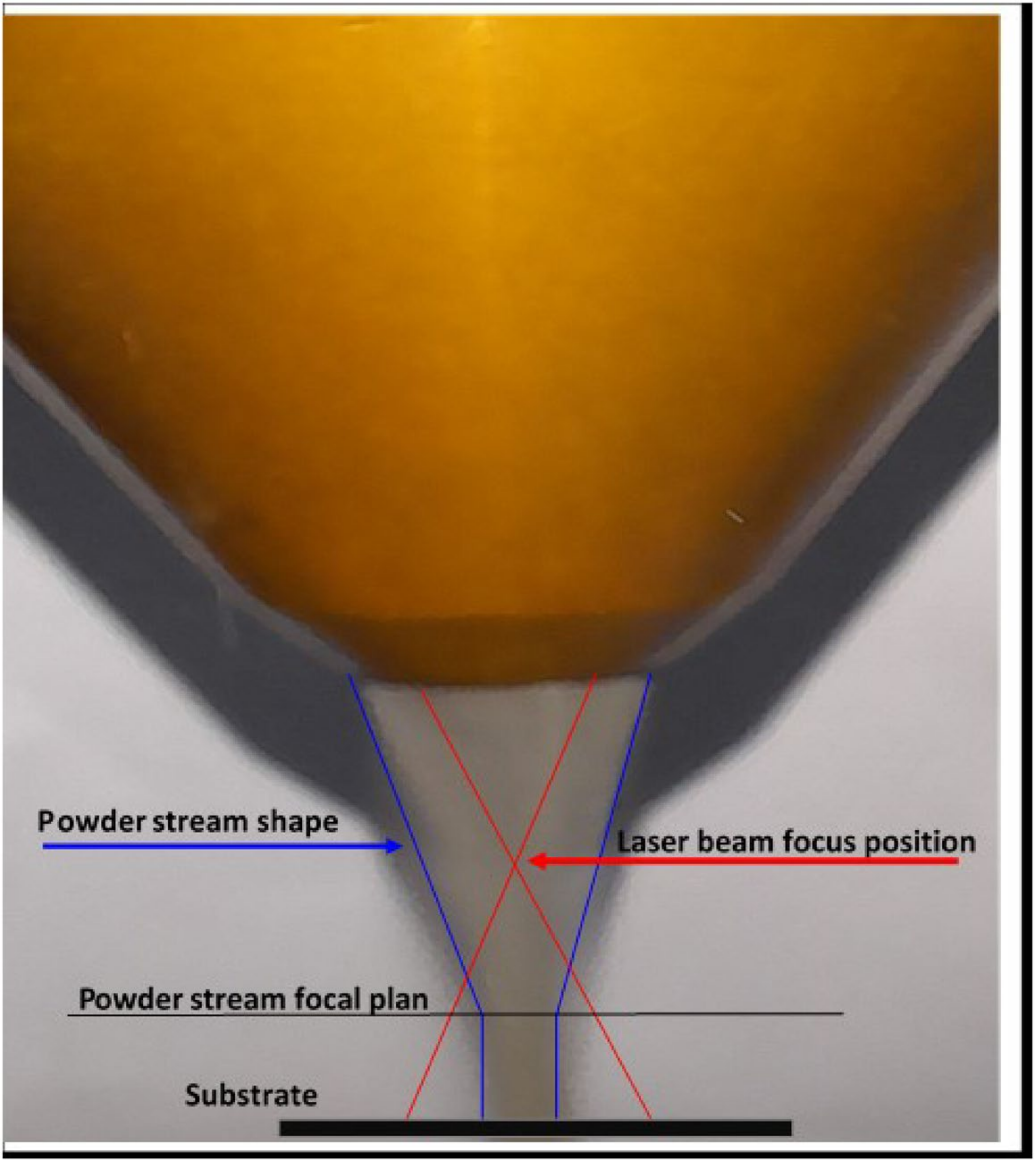
Illustration of the laser/powder stream planes adjustment.

At the same time, the laser focal plane is set at 3mm from the tip of the deposition head to increase the beam spot width of the incident laser beam to allow more interaction area with the powder stream. This step is significant as it determines the deposited bead width and the deposition process efficiency. Fig.7, shows the illustration of the laser/powder stream adjustment step. Based on this measurement, the laser spot size was set at 4mm to ensure that most of the powder particles were located inside the laser beam spot and to keep the intensity of laser energy at the maximum possible.

### Evaluation of the LMD process performance at maximum processing values

**Fig. 8:**
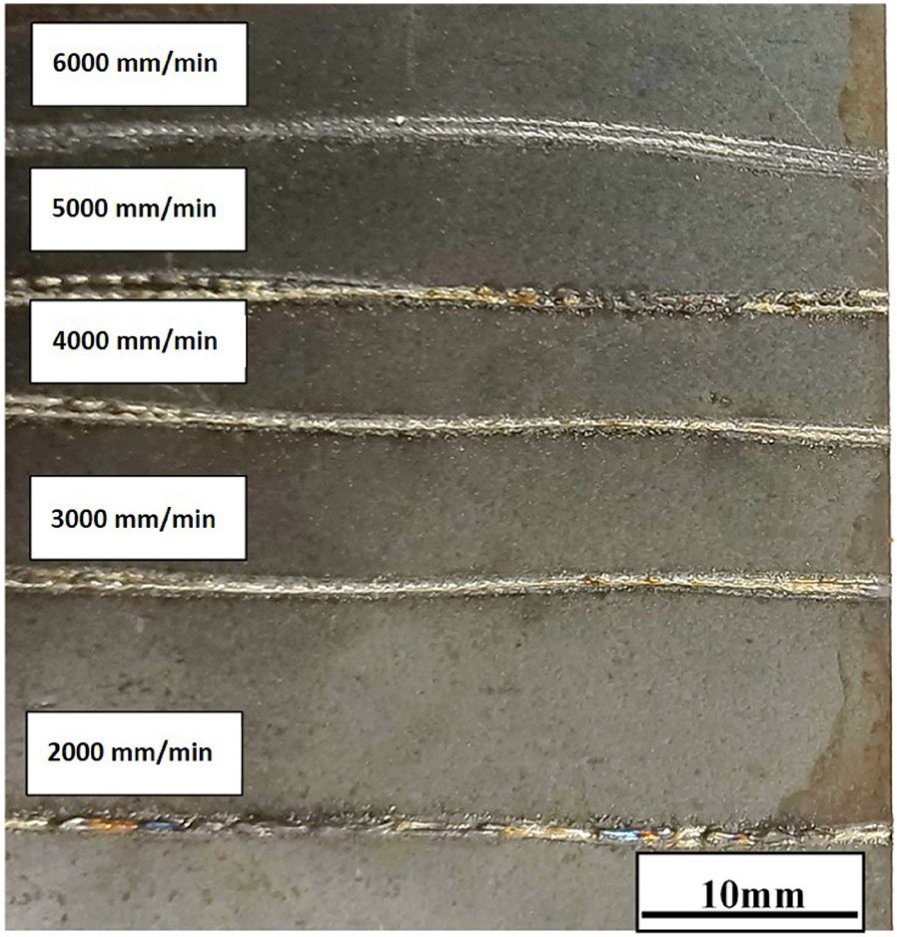
The bead shape changes with changing scanning speed at a powder feed rate 50 g/min and 3 KW laser power.

Increasing the productivity of the LMD process is vital, as it will reduce the necessary time required for the repair and/or protection process and, reduce all the process costs as well. To achieve this objective, the maximum value of the laser power and powder feed rate were used at various scanning speeds. In this regard, a complete laser power of 3KW was used for this series of experiments, as it is the total power that can be used with the available deposition head. In addition, the maximum powder feed rate of 50 g/min was used with that value of laser power at variable laser scanning speeds up to 8000 mm/min. At low scan speed, the powder particles were heated and melted without being attached to the substrate surface due to a large amount of powder (50 g/min) being deposited that restricted the laser beam from reaching the specimen surface even with high E and T as a result of using low scanning speed. Hence, a laser power over 3KW is required to use a significant powder feed rate value. By gradually increasing the scanning speed, the amount of deposited powder decreased a little and absorbed the incident laser beam, where the detached beads changed from spherical to slightly elongated shapes. Once the equilibrium point for the processing parameters is reached, where the E value is enough to melt the deposited powder at the proper T, a thin, well-adhesive bead is formed, as shown for scanning speeds of 2000 to 6000 mm/min. Specifically, slower scanning speeds (e.g., <2000 mm/min) result in wider beads due to increased energy density (E = P/(d$\times$v)) and longer interaction time (T = d/v), allowing more powder to melt and spread over a larger area. Conversely, higher scanning speeds (2000–6000 mm/min) produce narrower beads due to reduced E and T, limiting the melt pool size. Further increases in scanning speed result in too low E and short T that cannot melt the powder particles, and hence no bead is formed. Fig.8, shows the formed bead with a changing scanning speed from 2000 to 6000 mm/min at a 50 g/min powder feed rate, where below or above these values, no deposition or bad beads were obtained at these used parameters. Regardless of the quality of the beads, the results showed very narrow beads due to the E ranging between 10 to 30 J/$\hbox {mm}^2$, and the T ranging from 0.03 to 0.09 sec at a scan speed of 2000 to 6000 mm/min, allowing a small portion of powder to be melted to form thin wall beads. Therefore, a low scan speed below 2000 mm/min are necessary to obtain wide beads.

Fig.8, shows also a wavy shape that may be back to the motion of the robot system used in this study as it has six motors that may have a vibration influence on the movement. This waviness, observed as non-linear track profiles, is attributed to minor vibrations in the six-axis robotic system, which become more pronounced at higher scanning speeds (e.g., >1000 mm/min). Hence, all experiments were limited to less than 1000 mm/min scan speed. While the robotic system is mainly used for 3D repairing, this known issue limits its use for high scanning speed. To mitigate waviness in track profiles, CNC machines are recommended for high scan speeds (>1000 mm/min) in LMD, especially for 2D repairs, to ensure smoother and more linear bead deposition. Alternatively, a CNC machine is preferable as it can be used at a higher processing speed, especially for 2D repairing. The results showed that a laser beam power over 3 KW is needed to maximize the deposited bead width and height when a high powder feed rate (50g/min) is used at a low scanning speed. In comparison, a CNC machine is preferable to be used for over 1000 mm/min scanning speed for 2D repairing applications.

### Statistical analysis of process parameters

A two-way ANOVA with interactions was conducted to evaluate the effects of laser power (P), scan speed (v), and powder feed rate (F) on bead dimensions (width and height). These dimensions are fundamental to both surface coverage efficiency for protective coatings and material build-up rate for dimensional restoration and 3D printing. The analysis was performed using data from three replicates for each parameter combination, as outlined in table 5. The experimental design included the following parameter ranges:

- Laser Power (P): 1000 W, 2000 W, 3000 W
- Scan Speed (v): 1000 mm/min, 2000 mm/min, 6000 mm/min
- Powder Feed Rate (F): 10 g/min, 30 g/min, 50 g/min.

The ANOVA model included main effects (P, v, F) and two-way interactions (P*v, P*F, v*F). The obtained results are represented in tables 6 and 7. For bead width, laser power (F = 48.76, p < 0.001, Contribution = 43.7%) and powder feed rate (F = 38.84, p < 0.001, Contribution = 34.8%) were the dominant factors, followed by scan speed (F = 13.92, p = 0.002, Contribution = 12.5%). Higher power increases the energy density, leading to a larger melt pool and consequently a wider bead. In addition, a higher feed rate delivers more material to the melt pool, which, if fully melted, contributes to a wider bead. While low speeds produce wider beads, as scan speed increases, the laser interaction time at any given point decreases, resulting in a smaller melt pool and a narrower bead. Two-way interactions were not significant (p > 0.05), contributing 2.5–3.3% to the variance. This suggests that the combined effects of these parameters are minimal compared to their individual effects, likely due to the dominant role of energy density (E = P / (d $\times$ v)) in determining bead width. For bead height, scan speed was the most influential (F = 39.50, p < 0.001, Contribution = 37.9%), followed by powder feed rate (F = 30.75, p < 0.001, Contribution = 29.4%) and laser power (F = 24.00, p < 0.001, Contribution = 23.1%). A slower scan speed at a constant powder feed rate means more powder is deposited per unit length, leading to an increase in bead height. The laser power has a less pronounced direct effect on height compared to its effect on width, where its primary role is to ensure the powder is fully melted and adhered to the substrate. The P*v interaction shows a significant effect ( p = 0.068, Contribution = 5.4%), suggesting that the effect of laser power on bead height may vary slightly with scan speed, particularly at lower speeds where energy density is higher. P*F and v*F interactions are not significant (p > 0.05), with contributions of 3.8% and 3.4%, respectively. These results confirm that fine parameter tuning can optimize bead dimensions for specific applications, such as wide beads for corrosion protection or high beads for 3D repair.

**Table 6:** ANOVA snalysis for bead width.

Source	Sum of Squares (SS)	Degrees of Freedom (df)	Mean Square (MS)	F value	p value	Contribution (%)
Laser Power (P)	28.45	2	14.23	48.76	<0.001	43.7
Scan Speed (v)	8.12	2	4.06	13.92	0.002	12.5
Powder Feed Rate (F)	22.67	2	11.34	38.84	<0.001	34.8
P*v	2.15	4	0.54	1.85	0.161	3.3
P*F	1.98	4	0.5	1.71	0.188	3
v*F	1.62	4	0.41	1.4	0.272	2.5
Error	4.21	14	0.3			6.5
Total	65.08	26				100

**Table 7:** ANOVA analysis for bead height.

Source	Sum of Squares (SS)	Degrees of Freedom (df)	Mean Square (MS)	F-value	p-value	Contribution (%)
Laser Power (P)	1.92	2	0.96	24.00	<0.001	23.1
Scan Speed (v)	3.15	2	1.58	39.50	<0.001	37.9
Powder Feed Rate (F)	2.45	2	1.23	30.75	<0.001	29.4
P*v	0.45	4	0.11	2.75	0.068	5.4
P*F	0.32	4	0.08	2.00	0.141	3.8
v*F	0.28	4	0.07	1.75	0.191	3.4
Error	0.56	14	0.04			6.7
Total	8.32	26				100.0

### The LMD process optimization

The protection layers consisted of several parallel, overlapped beads. Generally, the single bead passes through three stages depending on the used level of the E. Stage one, is an interrupted bead where no deposition occurred due to improper processing parameters. In this stage, the powder particles were heated without being attached to the substrate surface due to using lower E . Stage two called the detached bead, where a bead is formed with balling defects. In this stage, with increasing the E, the globular melted metal increased in size and formed a detached bead which changed from spherical to slightly elongated shapes due to the enlarging of E. Stage three called the complete bead where the E is further increased to reach the optimum values where a sound bead is formed and overlapped with another bead to form a complete layer. The morphology of beads that form the three stages is represented in Table 8.

**Table 8:** Stages of bead formation in LMD, defined by energy density (E) and resulting morphology.

Stage	Description	Energy Density [J/mm$^2$]	Photo	Key Features
One	No deposition: Insufficient E to melt powder	<50		Powder scatters, no bonding
Two	Detached Bead: Partial melting, balling defects	50-100		Globular beads, weak substrate adhesion
Three	Attached Bead: Full melting, sound bead formation	100-200		Continuous, defect-free layer

The above-mentioned experiment showed that specimens consistently displayed poor visual quality when energy E dropped below 50 J/mm$^2$, regardless of powder feed rate or interaction time. This low E may result in insufficient melting or weak bonding, as seen in stages one and two of Table 8, suggesting that an E above 50 J/mm$^2$ is essential for satisfactory specimens. However, an E exceeding 200 J/mm$^2$ can cause overheating or porosity, degrading specimen quality, as shown in table 9. Thus, an intermediate E range of 50–200 J/mm$^2$ appears critical, yielding a mix of good and bad specimens. This indicates that powder feed rate and interaction time significantly influence results. Based on these results, fine-tuning of preferred parameters was made to get flexible and easily changed operation conditions to be applied according to the required applications. The processing scanning speed was determined to be between 250 mm to 1000 mm/min to be used with the robot. The laser power was set to range from 1.5 to 3 KW, and the powder feed rate was varied from 10 to 30 g/min. The deposited beads were evaluated visually and defined as good or bad as shown in table 9, according to the bead shape where the bad shape may have some porosity, in complete or having balling solidified metal. while good results that show a continuous and smooth bead.

**Table 9:** Visual evaluation examples of the deposited beads.

Evaluation	Description	Photo
Good	Wide bead, no visual defects	
Good	Narrow bead, no visual defects	
Bad	Porosities	
Bad	Balling effect	
Bad	No deposition	
Bad	Detached bead	
Bad	Overheating	

**Fig. 9:**
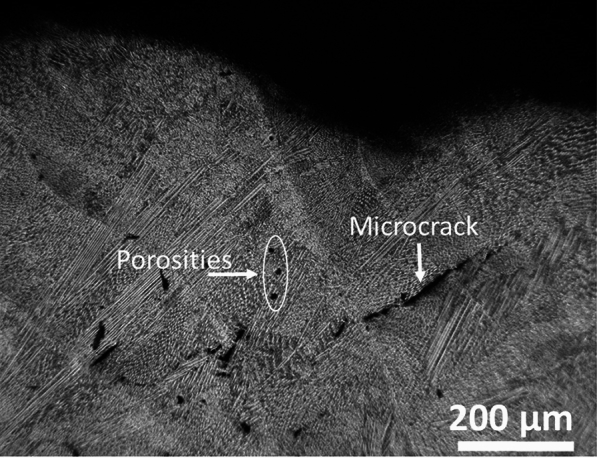
The porosities and microcracks appear at improper process parameter combinations.

Generally, the resulting bead shape depends on the proper combination of the LMD process parameters. As the E increased, more powder melted in the molten pool, and spread to cover a wide area with an increasing T. Also, with increasing the F the bead width increased due to a large portion of incident metal powder absorbing the laser energy and spreading over a wide area. Similar observations are reported in^16^. Increasing E and F with a short T led to diminishing the bead width but increasing the bead height. Similar observations have been reported^15^. While too long T and high F resulted in beads with porosities or microcracks as shown in Fig.9. According to these results, the optimum range of F that can be used in this study was 10 to 30 g/min at E ranging from 100 to 200 J/mm$^2$. The low F below 10 g/min yield a thinner bead that not is preferable for industrial applications and is not economically wise, where no good results were obtained over 30 gm/min due to the limited E that depends on the available laser power source (3KW). The effective T that yielded good results with the used value of E and F was found to be between 0.5 to 1.6 sec. Accordingly, different deposited bead widths ranged from 1 mm to 4 mm, and heights ranging from 1 mm to 3 mm were obtained. The optimum processing parameters come in agreement with the used parameters in previous studies for the same materials in^5,6^. The order of controlling or dominated parameters is E then T then F. It is clear that wide beads need a high E associated with a high F and a long T and vice versa. Hence, the optimum processing window helps to select the best parameters according to the required applications. It is worth mentioning that the bead height is inversely proportional to bead width, where the lowest height is at the wider bead. Accordingly, for surface protection applications against corrosion, it is preferable to use a wider bead. While for rebuilding damaged parts and 3D printing applications, using the high bead is recommended.

### Layer microstructure

**Fig. 10:**
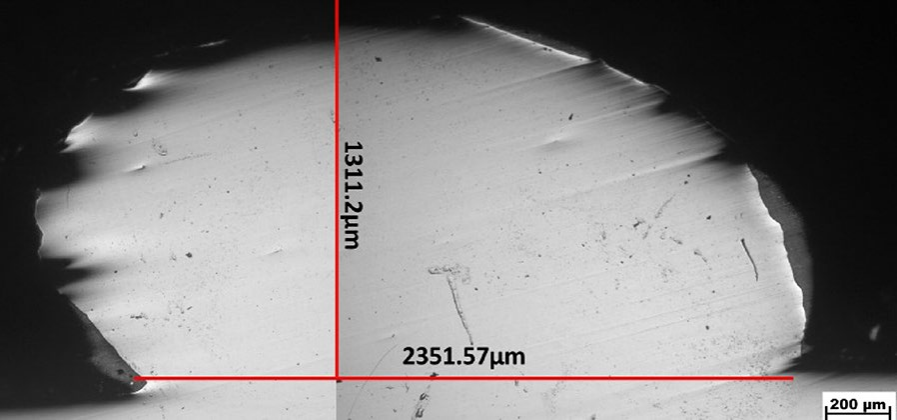
Single bead cross-section with width and height.

A typical cross-section of the deposited single bead of the optimum specimen at E (140 J/mm$^2$) , T(0.96s) and F (30 g/min) is shown in Fig.10 with its main width and height.

**Fig. 11:**
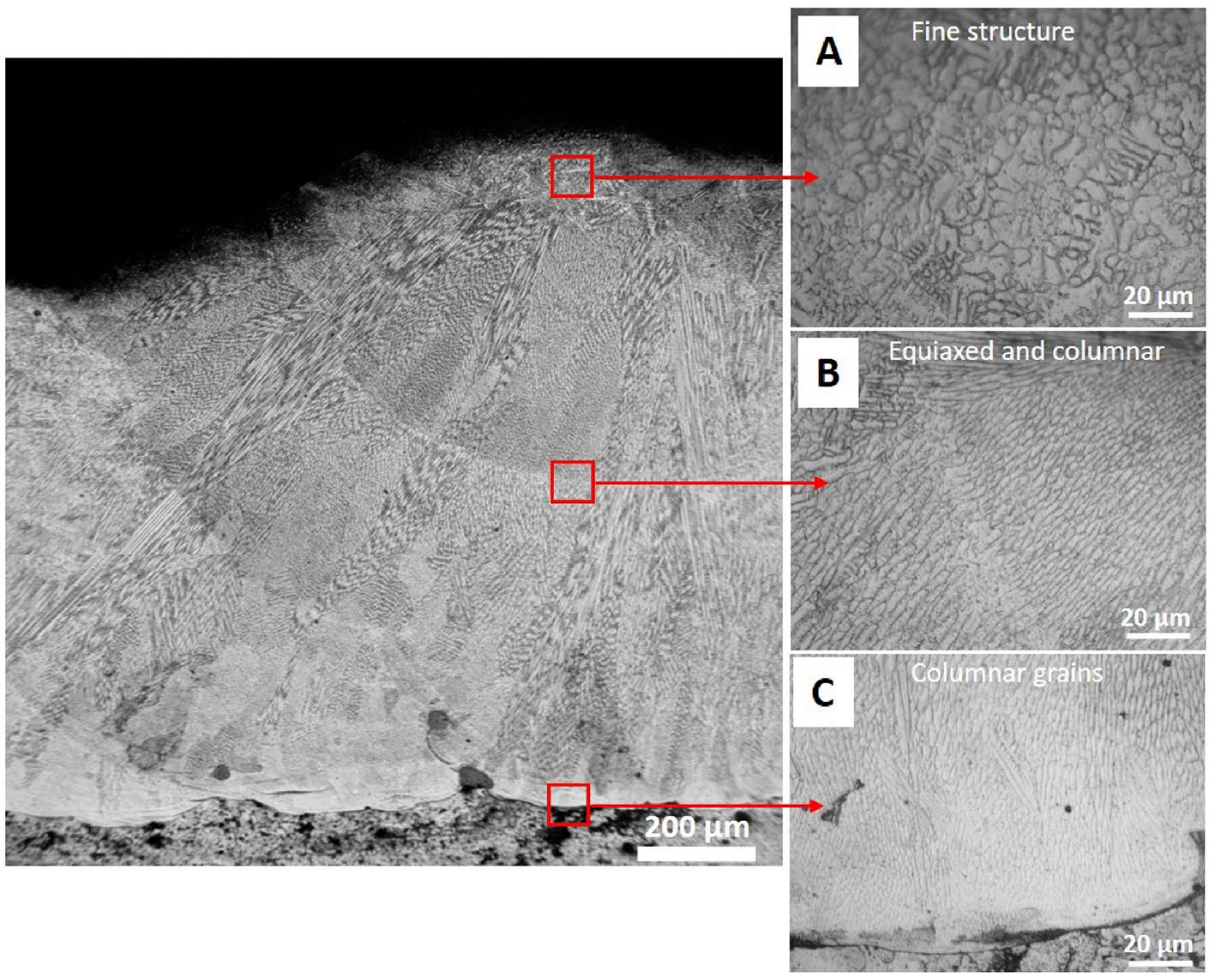
Overview of the microstructure of the deposited 304L stainless steel layer, (**a**) fine structure at the top of the layer, (**b**) equiaxed and columnar grains at the center, (**c**) columnar grains at the interface.

In addition, the layer is free from defects such as microcracks, and the lack of fusion with observed some fine porosities. Fig.11, shows a typical morphology of solidification structures, free from defects such as microcracks, and the lack of fusion with observed some fine porosities indicating that the metallurgical bonding between the layer and the substrate was excellent. The microstructure showed cellular, equiaxed, and columnar dendritic growth of several grains from the interface zone developing toward the direction of heat transfer, which is typical for LMD parts^6,33,38,45,46^. The formation of fine grains due to the rapid heating and solidification process that occurred in the LMD process may improve the mechanical performance as it reduces the microcracks susceptibility, which agrees with the previous investigations^13,33,45,46^.

**Fig. 12:**
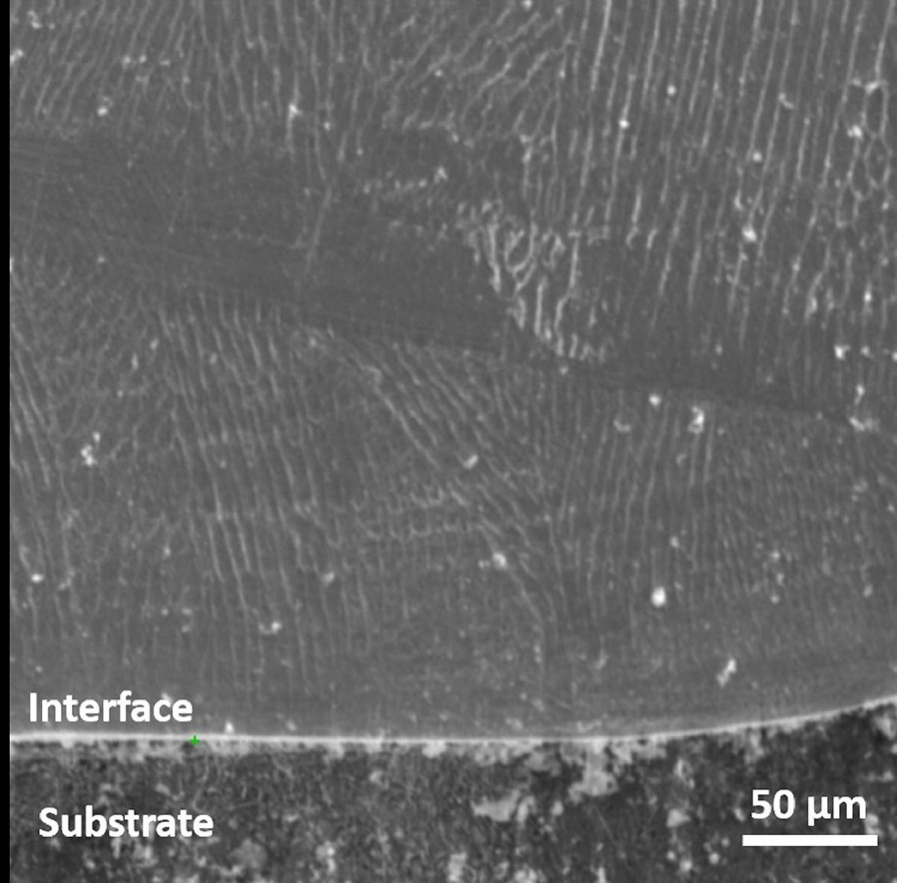
SEM of the interface zone.

**Fig. 13:**
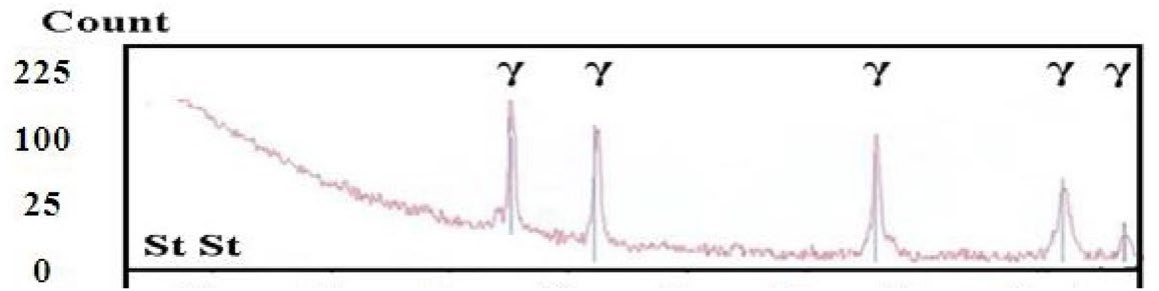
XRD of the deposited 304L stainless steel layer.

The SEM image of the deposited layer, presented in Fig.12, reveals a distinct and robust interface between the substrate and the layer, with no visible cracks or pores due to the high cooling rate that characterizes the LMD process^13,34^. XRD analysis was performed to identify the resulting phases. As shown in Fig.13, only a strong peak corresponding to the austenite phase was observed for stainless steel 304L, indicating that no phase changes occurred in the deposited layer. The austenite phase’s persistence after solidification can be attributed to the Cr/Ni ratio in stainless steel 304L combined with a high cooling rate.

The different sizes and morphologies of the microstructure depend on the combination of the temperature gradient (G) and the solidification velocity (R) through the Eqs. (3 and 4)^33,45,46^:

3 $$\begin{aligned} S = G . R\quad K/s \end{aligned}$$

Where, (S) is the cooling rate which is usually in the range between 10$^2$ -10$^4$ K/s for the LMD process, and higher than that of the conventionally manufactured processes^5,33,45,46^. At such a high cooling rate, a fine structure is formed^5,33,45,46^ which in turn improved the hardness and corrosion resistance of the 304L stainless steel layer as shown later in this study. The microstructure morphology (M) is determined by eq.4:

4 $$\begin{aligned} M = G/R \end{aligned}$$

Depending on G/R, different morphology such as planner, columnar, cellular, and dendritic can exist as shown in Fig. 10. At the beginning of the process, the ratio of G/R was high, with a large G and an extremely slow R which resulted in the formation of planar grain at the bottom of the molten pool. Then G gradually decreases while R gradually increases, which results in the decrease in the ratio of G/R, from the bottom to the top of the molten pool, leading to columnar dendrite growth followed by cellular to dendritic structures that characterize the LMD process. Meanwhile, fine equiaxed grains formed at the top of the layer due to the contact with the surrounding atmosphere, which increased heat dissipation through convection and radiation. In addition, the interface between the layer and substrate is fine due to the high cooling rate that characterizes the LMD process. Furthermore, the observed heat-affected zone (HAZ ) is very thin.

**Fig. 14:**
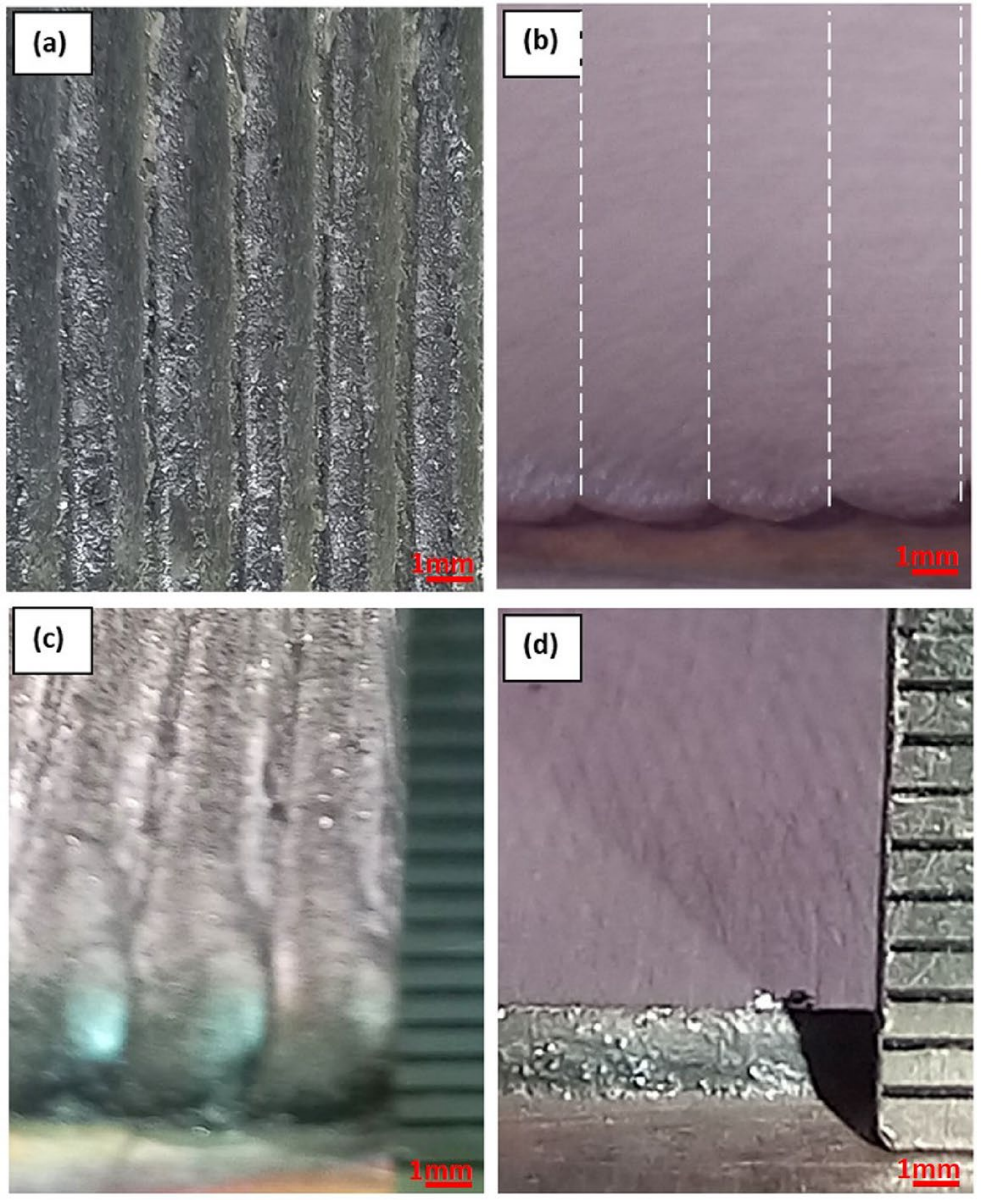
(**a**) Top view of the as-deposited layer, (**b**) top view of the polished layer with bead boundary (dashed line), (**c**) side view of the as-deposited layer thickness, (**d**) side view of the polished layer thickness.

The optimum processing parameters that resulted in a wide bead shape were used to deposit a complete protective layer through overlapped beads as shown in Fig.14. Due to the overlapped beads, a wavy surface appeared on the top of the deposited layer.

The wavy surface is undesirable in industry and needs post-processing machines such as grinding and /or polishing to obtain a smooth surface. Besides the surface waviness, it is helpful to consider the height of the deposited layer after grinding, which is known as adequate layer thickness.

### Microhardness measurements

**Fig. 15:**
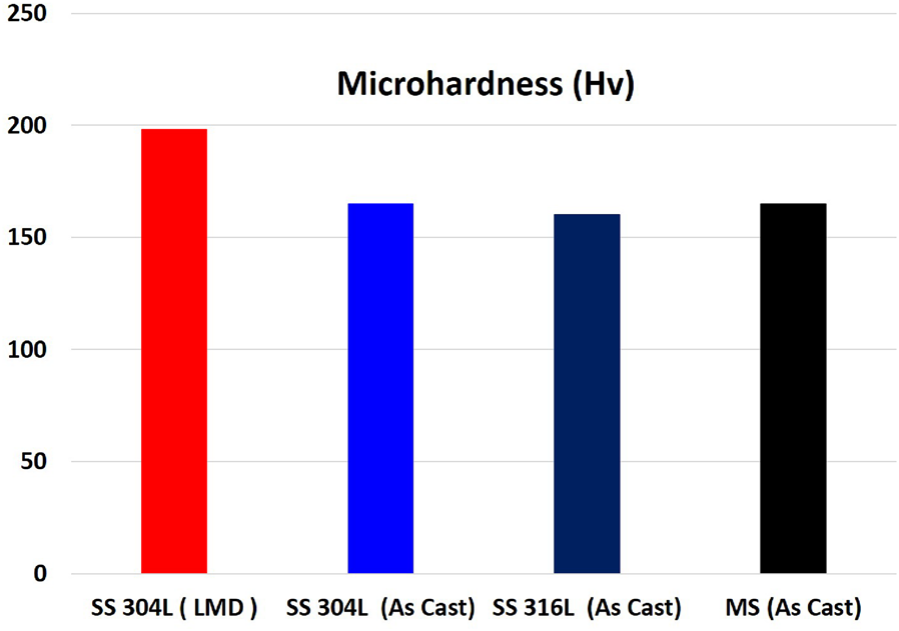
Microhardness measurements of the deposited 304L layer, as cast 304L, 316L and mild steel.

The value of the average microhardness measurements using Hv(0.3kg) of the deposited layer of the selected specimen made at E (140 J/mm$^2$), T(0.96s) and F (30 g/min) was compared with as-cast 304L,316L stainless steel and mild steel and represented in Fig.15.

The hardness of the deposited layer was around 200HV which is larger than the other materials which in good agreement with previous studies^7,18,38^.

The increase in hardness values of the deposited layer referred to fine grains, which agrees with the observed microstructure shown in Fig.11. This result is in good agreement with similar observations reported in^7,38^. It is worth mentioning, that the obtained hardness should provide excellent behavior to both 316L stainless steel and mild steel engineering components in the field service.

However, extreme hardness values are usually obtained by reinforcing the deposited layer with ceramic materials such as WC and SiC for extra protection in extreme field services, as reported in^45–47^. Though reinforcement ceramic materials provide high performance, they, on the other hand, increase the overall cost of the LMD process and may cause a series of damage to the other engineering components that are in contact with the repaired parts. Thus, it is preferable for repairing applications to use a similar material to the base metal or material that has relatively the same properties, which is achieved in this study.

### Corrosion behavior

**Fig. 16:**
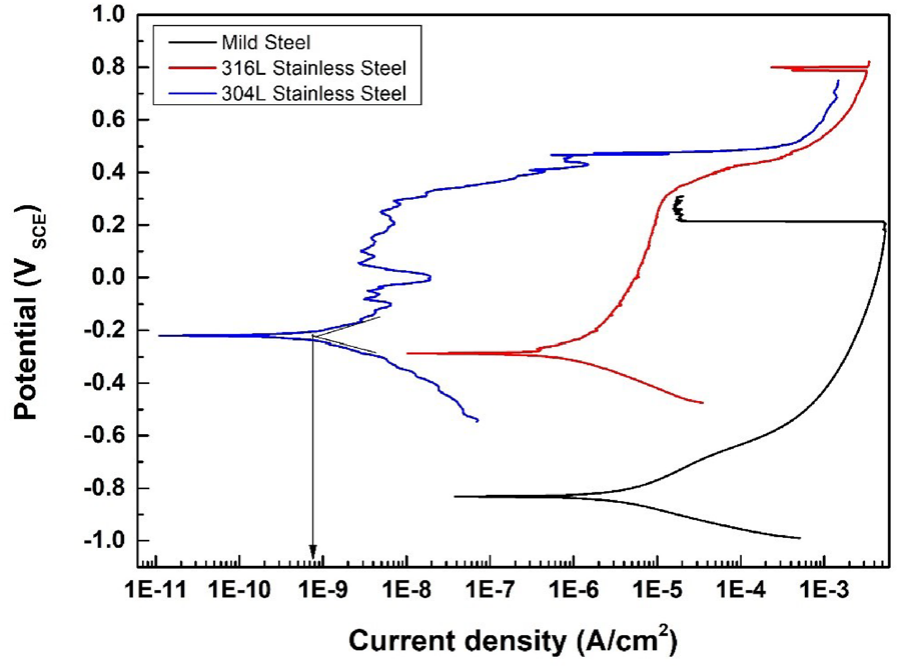
Potentiodynamic polarization curves for 304L stainless steel layer, 316L stainless steel, and mild steel substrates.

Fig.16 presents potentiodynamic polarization curves for the 304L stainless steel layer compared to untreated 316L stainless steel and mild steel substrates.

The corrosion current density $\hbox {i}_{corr}$ was calculated by extrapolating the rectilinear Tafel segments of the anode and cathode polarization branches, and the polarization resistance (Rp) provided the corrosion rates (CRs) from the Stern–Geary equation^48^ listed in Table 10. The potential of both the 304L stainless steel layer and 316L stainless steel substrate shifted in a noble direction compared to the mild steel substrate. This observation could be related to a higher percentage of Cr and Ni content compared to the mild steel as presented in table 4.

The dissolved Cr and Ni may form stable chromium and nickel oxide passive films on the layer surface which is known for their high corrosion resistance^5,16,49,50^. Furthermore, while the 304L stainless steel layer and 316L stainless steel substrate revealed almost the same potential, a huge gap in the corrosion current between them was observed. This gap may attributed to the grain refining of the 304L stainless steel layer as shown previously in Fig.11, due to the heating and cooling effect in the LMD process, where fast heating and high cooling rate led to fine grain size compared with the base metal where the fine grains increase grain boundary density, promoting stable passive film formation. Similar behavior was observed and reported elsewhere^51^.

**Table 10:** Potentiodynamic data for 304L stainless steel layer, 316L stainless steel and mild steel substrates.

Specimens	$\hbox {E}_{\text {corr}}$ (VSCE)	$\hbox {i}_{\text {corr}}$ ($\mu$A/$\hbox {cm}^2$)	C.R (mpy)
Mild steel substrate	-0.83	8.6	0.067
316 L substrate	-0.28	1.5	0.012
304 L layer	-0.22	0.02	0.002

These results showed the possibility of producing a smooth, well-adherent and high corrosion resistance 304L layer on AISI 316L stainless steel and mild steel substrates for surface protection and repairing of worn parts which may extend their usage in various applications in industry.

### Cost-effectiveness analysis

**Fig. 17:**
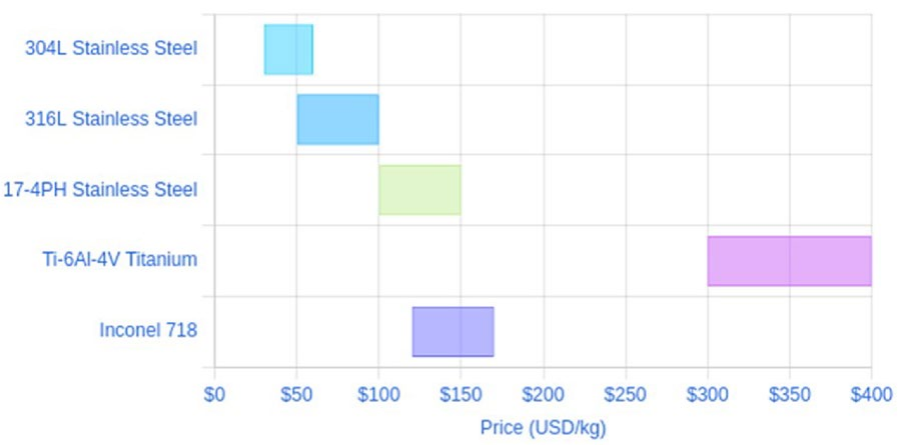
Metal Powder Prices for AM (USD/kg)^55–57^.

Stainless steel 304L and 316L are two widely used austenitic grades in additive manufacturing (AM). While both offer excellent corrosion resistance and mechanical properties, their material costs differ significantly. The 304L is a low-carbon ($$\le$$0.03% C) version of 304 stainless, containing approximately 18–20% chromium and 8–12% nickel. It is the most common austenitic stainless steel in AM due to its good all-around corrosion resistance and weldability. The low carbon content minimizes carbide precipitation during welding/fusing, enhancing resistance to intergranular corrosion. 304L parts exhibit high strength, excellent ductility, and good corrosion resistance in many environments^52,53^,. It is generally less expensive than 316L, making it a cost-effective choice for applications that don’t require the superior corrosion resistance of 316L. On the other hand, 316L is also a low-carbon grade ($$\le$$0.03% C), but it contains $$\sim$$16–18% chromium, 10–14% nickel, and an addition of 2–3% molybdenum. The molybdenum gives 316L greater resistance to pitting and crevice corrosion in chloride-rich environments^54^. Hence, 304L is generally chosen for its balance of performance and lower cost, whereas 316L is chosen for its enhanced corrosion resistance in demanding environments. However, because 316L contains more nickel and molybdenum, the raw powder is costlier which directly increases powder production cost. Therefore, 304L is generally the more economical choice. Industry reports indicate that 304L powder prices can range from roughly $30/kg up to $50–60/kg for typical AM-grade material, with smaller orders or finer powders on the higher end of that range. While 316L powder prices tend to start around $50/kg and go up to $80–$100/kg for typical AM usage, which is generally higher than 304L’s price range. The exact cost varies but is often on the order of 20–50% depending on the supplier and quantity. Fig.17 illustrates the typical price ranges for various metal powders used in AM , highlighting that 304L is generally the most economical stainless steel powder, followed by 316L. When using LMD to repair or manufacture industrial components, material cost is one of the key cost drivers (along with machine time, post-processing and labor). The cost difference between 304L and 316L can have a significant impact on the economics of LMD production, especially for large components or high material usage. Therefore, for cost-sensitive applications where 304L’s properties are adequate, choosing 304L can yield material cost savings. In addition, the cost impact of powder waste is greater for 316L, which makes the operators more diligent about recycling 316L powder to avoid wasting the more costly material. Unlike prior LMD studies^6,7,14,17^, which primarily used 316L stainless steel powder at lower deposition rates (<30 g/min), this work demonstrates the successful high-deposition-rate (up to 50 g/min) use of cost-effective 304L powder. This approach achieves defect-free layers with strong metallurgical bonding on both 316L and mild steel substrates, as evidenced in the microstructure and corrosion measurement sections. The deposited 304L layers exhibit comparable or superior mechanical properties (e.g., microhardness around 200 HV) and corrosion resistance (0.002 mpy in 3.5% NaCl) relative to 316L, while offering a 20–30% reduction in material costs. By addressing both economic and performance challenges, this work establishes a novel, industrially viable strategy for repairing and enhancing engineering components, distinguishing it from existing literature focused on higher-cost materials and lower deposition efficiencies.

## The LMD process risk observation

**Fig. 18:**
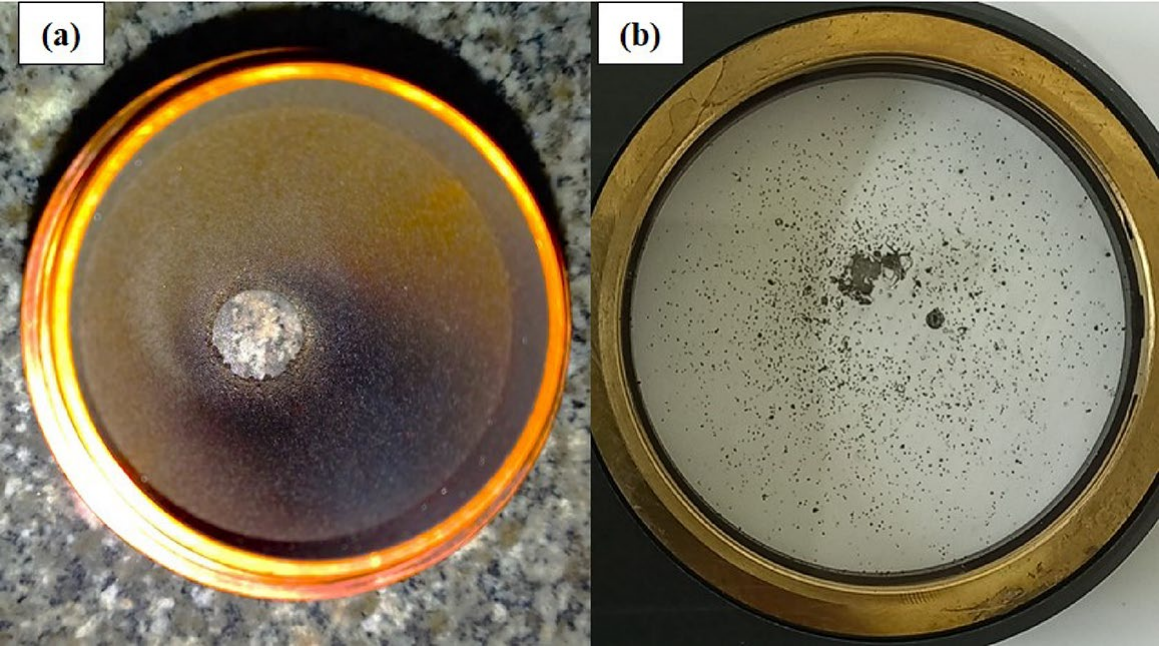
The influence of volatile powder and fumes (**a**) damaged nozzle (**b**) damaged protection glass.

The implementation of high-deposition-rate laser metal deposition (LMD) processes, as explored in this study, presents practical challenges that are critical to achieving the objectives of reliable and cost-effective repair of engineering components. Ensuring operator safety and equipment integrity is paramount for industrial adoption of LMD using 304L stainless steel powder at deposition rates up to 50 g/min. One significant risk observed during experimentation is powder adhesion to the deposition head, as illustrated in Fig.18. (a,b). This issue, caused by the accumulation of unmelted or partially melted 304L powder particles on the protective glass of the laser head, can obstruct the laser beam, leading to inconsistent energy delivery. Such inconsistencies directly affect bead quality, resulting in defects such as porosity or incomplete fusion, which compromise the metallurgical bonding and corrosion resistance reported in the microstructure and corrosion measurement sections. Additionally, powder adhesion can reduce process stability, increasing the likelihood of interruptions and raising operational costs due to downtime or rework. To mitigate these risks, regular maintenance of the LMD equipment is essential. For instance, cleaning the protective glass after every 1 hour of continuous operation at high deposition rates (e.g., 50 g/min) prevents powder buildup and ensures consistent laser focus. Furthermore, optimizing the shielding gas flow (maintained at 5 L/min in this study) minimizes powder scattering and adhesion. These measures enhance process efficiency and maintain the defect-free layer quality critical for cost-effective repairs. By addressing these practical challenges, this study supports the industrial viability of high-deposition-rate LMD, aligning with the objective of delivering reliable, economical solutions for repairing 316L stainless steel and mild steel components in applications such as aerospace and oil and gas industries.

## Conclusion

This study successfully demonstrates a cost-effective Laser Metal Deposition (LMD) process using 304L stainless steel powder, offering a high-deposition-rate solution for repairing and enhancing engineering components with superior performance and economic benefits. The approach not only addresses industrial needs for durable, precise repairs but also sets a foundation for scalable applications in challenging environments.

1. Defect-free 304L stainless steel layers were deposited on 316L stainless steel and mild steel substrates using Laser Metal Deposition (LMD) at high deposition rates (up to 50 g/min), achieving strong metallurgical bonding with minimal porosities and no microcracks.
2. Optimal parameters (energy density: 100–200 J/mm$^2$, interaction time: 0.5–1.6 s, powder feed rate: 10–30 g/min) ensured high productivity and process stability, reducing processing time and energy consumption.
3. The 304L layers exhibited higher microhardness (around 200 HV) compared to 316L and mild steel substrates, due to fine grain formation from rapid solidification.
4. The 304L layers showed corrosion resistance comparable to 316L (0.002 mpy) and superior to mild steel (0.067 mpy) in 3.5% NaCl, attributed to high Cr/Ni content and grain refinement.
5. Using 304L powder, approximately 20–30% cheaper than 316L due to the absence of molybdenum, combined with high deposition efficiency, reduced material and operational costs compared to 316L-based LMD or conventional methods.
6. High deposition rate LMD requires stringent safety measures, such as regular cleaning of the deposition head to prevent powder adhesion, ensuring consistent layer quality and equipment integrity.
7. The process enables repair and protection of components like pump shafts, valve bodies, and hydraulic cylinders in industries such as water treatment, chemical processing, and heavy machinery, extending service life and reducing costs.
8. The LMD offers superior precision and lower thermal distortion compared to wire arc additive manufacturing (WAAM), making it ideal for sensitive component repairs.

## Future work

To further advance the cost-effective LMD process using 304L stainless steel powder for repairing and enhancing engineering components, the following investigations are proposed:

1. Conduct tensile, residual stress analysis using XRD, and wear tests to fully evaluate the repaired components’ ductility, fatigue life, and wear resistance for industrial applications.
2. Test 304L layers in acidic (e.g., $\hbox {H}_{2}$ $\hbox {SO}_{4}$) and marine environments to validate corrosion performance for chemical processing and naval applications.
3. Apply LMD to titanium and nickel superalloys, optimizing parameters to achieve defect-free layers for aerospace and high-temperature uses.
4. Investigate LMD scalability for large components and in-situ repairs, improving robotic/CNC precision and process stability. These efforts will enhance the industrial applicability of the LMD process, ensuring its reliability, cost-effectiveness, and versatility for a wide range of engineering components and environments..

## Data Availability

The datasets used and/or analysed during the current study are available from the corresponding author on reasonable request.
